# Quantitative Assessment of Respiratory-Induced Clinical Target Volume Motion During Postoperative Whole-Breast Radiotherapy Using Four-Dimensional Computed Tomography

**DOI:** 10.3390/cancers18132142

**Published:** 2026-07-02

**Authors:** Ji Hwan Jo, Jeong Won Lee, Ki Ho Seol

**Affiliations:** 1 Department of Radiation Oncology, Daegu Catholic University Medical Center, Daegu 42472, Republic of Korea; d240063@cu.ac.kr (J.H.J.); wonie1016@naver.com (J.W.L.); 2 Department of Radiation Oncology, Daegu Catholic University School of Medicine, Daegu 42472, Republic of Korea

**Keywords:** breast cancer, respiratory motion, 4DCT, CTV displacement, internal margin, postoperative radiotherapy, breast-conserving surgery

## Abstract

**Background/Objectives:** We quantitatively evaluated respiratory-induced clinical target volume (CTV) motion during postoperative whole-breast radiotherapy in patients undergoing breast-conserving surgery (BCS) using four-dimensional computed tomography (4DCT) and determined the internal margins for assessing respiratory motion. **Materials and Methods:** We enrolled 100 patients who underwent postoperative whole-breast breast RT after BCS. Simulation CT was performed using 4DCT, and the CTV was delineated for the 10 respiratory phases (0–90%). CTV center displacement was measured in the lateral, anteroposterior, and superoinferior directions and three-dimensional (3D) vector magnitudes were calculated. Internal margins for respiratory motion were calculated using the Van Herk formula (2.5Σ + 0.7σ). **Results:** Mean CTV center displacement (mm) was 0.94 ± 0.52 (lateral), 1.29 ± 0.59 (anteroposterior), and 1.00 ± 0.51 (superoinferior). The mean 3D vector magnitude was 1.99 ± 0.68 (range, 0.70–3.51) mm. Anteroposterior motion was significantly greater than the lateral and superoinferior motion (*p* < 0.001). No patient exhibited motion exceeding 3 mm in any direction. Right-breast 3D motion was significantly larger than the left-breast motion (2.17 ± 0.66 vs. 1.82 ± 0.65 mm, *p* = 0.008). Internal margins (mm) for respiratory motion were 1.02 (lateral), 1.24 (anteroposterior), and 1.03 (superoinferior). **Conclusions:** Respiratory-induced CTV motion during whole-breast RT after BCS was small, with anteroposterior dominance. Calculated internal margins were approximately 1 mm in all directions, suggesting that a conventional 5 mm CTV-to-planning target volume (PTV) margin is adequate to compensate for respiratory motion and indicating that routine 4DCT or respiratory motion management may be unnecessary for target coverage.

## Simple Summary

Breast cancer patients who undergo breast-conserving surgery typically receive postoperative radiation therapy to reduce the risk of local recurrence. During radiation therapy, breathing causes the breast to move slightly, which could potentially affect the accuracy of radiation delivery. This study aimed to quantify breathing-induced breast motion using four-dimensional computed tomography, which captures target movement across the full respiratory phase. We found that respiratory motion was consistently small in all directions, with mean displacement under 1.3 mm and no patient exceeding 3 mm in any direction. Patients with right-sided tumors and lower body mass index showed slightly greater motion. Our findings suggest that a conventional 5 mm planning margin is sufficient to compensate for respiratory motion, and that dedicated respiratory motion management techniques are not routinely necessary for target coverage in postoperative whole-breast radiation therapy.

## 1. Introduction

Postoperative radiotherapy (RT) following breast-conserving surgery (BCS) is an essential component of breast cancer treatment, providing significant improvements in local control, and long-term follow-up studies support the oncological safety of breast-conserving approaches [1,2]. With advances in treatment planning and delivery technologies, intensity-modulated RT (IMRT) has become a widely used technique for whole-breast irradiation in many institutions, replacing conventional three-dimensional conformal RT (3D-CRT) because of its superior dose conformity and homogeneity [3,4,5].

Accurate dose delivery in RT depends on precise knowledge of the target position throughout the treatment course. Respiratory motion introduces geometric uncertainties that can affect treatment accuracy by displacing the target volume relative to the planned position. Respiratory motion is a well-recognized source of significant geometric uncertainty in thoracic and abdominal malignancies such as lung and liver cancers, and four-dimensional computed tomography (4DCT) is routinely employed for treatment planning to account for this motion [6,7,8]. However, in breast cancer RT, respiratory-induced target motion is generally considered smaller than that observed in thoracic or abdominal targets, and conventional CT acquired during free breathing has commonly been used for whole-breast simulation and planning [9,10].

Although this assumption is widely accepted, the quantitative evidence supporting it remains limited, particularly in the context of modern IMRT delivery. Unlike 3D-CRT, where tangential fields inherently provide generous coverage of the breast tissue, IMRT utilizes steep dose gradients and complex beam modulation that may be more susceptible to the effects of target motion, including the interplay between the moving target and the dynamic multileaf collimator [11,12,13]. Furthermore, as hypofractionated regimens become increasingly prevalent [14], understanding the dosimetric impact of motion remains clinically relevant [15].

Several studies have investigated respiratory motion in breast cancer RT, but each was limited in scope. Wang et al. used free-breathing 4DCT to correlate respiration-induced breast target motion with dosimetric variance, reporting centroid displacements on the order of 1 mm, but in a small cohort of 17 patients [16]. In a companion study, the same group compared dosimetric variance between 4DCT- and 3DCT-based whole-breast forward-planned IMRT, again limited to 17 patients [17]. Qi et al. evaluated respiration-induced cardiac motion to derive gating indications, but their analysis was confined to 20 left-sided patients and focused on the heart rather than the breast clinical target volume (CTV) [18]. Lowanich-kiattikul et al. assessed chest-wall motion primarily under breath-hold conditions rather than across the free-breathing cycle [19]. Collectively, prior work has been constrained by small sample sizes, evaluation of few respiratory states, or a focus on selected settings such as left-sided treatment or breath-hold-based techniques. A comprehensive quantitative assessment of CTV motion across the full respiratory cycle in a large patient cohort is therefore lacking.

The purpose of this study was to quantitatively evaluate respiratory-induced CTV center displacement during postoperative whole-breast RT in patients who underwent BCS, to identify patient characteristics associated with motion magnitude, and to calculate the internal margin attributable to respiratory motion using the Van Herk formula.

## 2. Materials and Methods

### 2.1. Study Population

**Figure 1:**
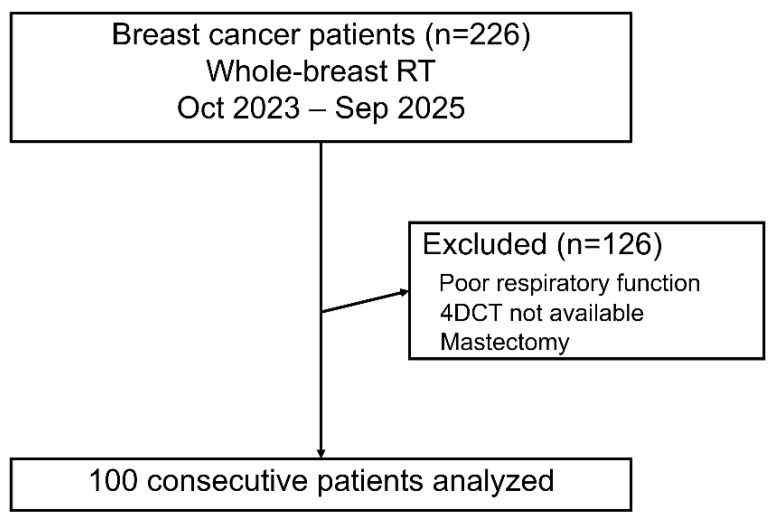
Flowchart of patient enrollment.

Between October 2023 and September 2025, a total of 226 patients with breast cancer underwent postoperative whole-breast RT at our institution. Of these, 126 were excluded because of poor respiratory function, unavailability of 4DCT simulation data, or treatment by mastectomy with chest-wall irradiation, leaving 100 consecutive patients for analysis (Figure 1). The inclusion criteria were: (1) histologically confirmed breast cancer treated with BCS; (2) planned postoperative whole-breast RT; and (3) availability of 4DCT simulation data.

The study protocol was reviewed and approved by the Institutional Review Board (IRB) (IRB number: 2026-03-044). The requirement for patient consent was waived due to the retrospective nature of the study and the use of anonymized data.

### 2.2. 4DCT Simulation

All patients underwent CT simulation in the supine position with both arms raised above the head, using a wing board for immobilization. Before the scan, patients were instructed to breathe regularly and shallowly to minimize respiratory motion artifacts. The 4DCT scan was performed using a Discovery 590 RT scanner (GE Healthcare, Waukesha, WI, USA) during free breathing. Respiratory signals were recorded using the deviceless 4D system on a GE Discovery 590 RT CT scanner. The acquired CT images were retrospectively sorted into 10 respiratory phases (0–90%, where 0% represents end-inspiration and 50% represents end-expiration) using Advantage 4D software version 1.0.36 (GE Healthcare, Waukesha, WI, USA). Images were reconstructed at a slice thickness of 3.75 mm. Because image sorting relied on the deviceless 4D system rather than an external respiratory-waveform device, a quantitative external breathing trace was not recorded.

### 2.3. Target Delineation and Motion Measurement

The 4DCT image sets were transferred to the Eclipse treatment planning system (Varian Medical Systems, Palo Alto, CA, USA). The CTV, defined as the entire ipsilateral breast tissue, was delineated in each of the 10 respiratory phases by a single radiation oncologist in accordance with the Radiation Therapy Oncology Group guidelines.

The coordinates of the CTV center (center of mass) were automatically calculated using the Eclipse system for each respiratory phase. The total amplitude of CTV center displacement was determined as the peak-to-peak distance (maximum minus minimum position) across all 10 phases in each of the three orthogonal directions: lateral (LR, *X*-axis), anteroposterior (AP, *Y*-axis), and superoinferior (SI, *Z*-axis). The 3D vector magnitude was calculated using the Euclidean distance:

3D vector = √(*X*^2^ + *Y*^2^ + *Z*^2^)

### 2.4. Internal Margin Calculation

The internal margin attributable to the respiratory motion was calculated using the Van Herk formula [20]:

Margin = 2.5Σ + 0.7*σ*

where Σ represents the systematic error component, and σ represents the random error component.

This formula ensures that 90% of the patients receive a minimum cumulative CTV dose of at least 95% of the prescribed dose. The peak displacement from the mean position was estimated to be half the total amplitude (amplitude/2). The systematic error (Σ) was defined as the standard deviation (SD) of individual peak displacements across the patient population, reflecting the inter-patient variability in mean breathing position. The random error (σ) was defined as the root mean square of individual peak displacements, representing the intrafractional motion variability.

### 2.5. Statistical Analysis

Descriptive statistics, including the mean, standard deviation (SD), median, range, and interquartile range (IQR), were calculated for the CTV center displacement in each direction and for the 3D vector magnitude. The normality of each variable was assessed using the Shapiro–Wilk test. The directional displacement components (lateral, anteroposterior, and superoinferior) and BMI deviated significantly from a normal distribution (Shapiro–Wilk, *p* < 0.05); accordingly, non-parametric tests were used for the corresponding analyses, and both mean ± SD and median [IQR] are reported to fully characterize the distributions. Pairwise comparisons of motion amplitude between directions were performed using the Wilcoxon signed-rank test, which is appropriate for paired, non-normally distributed measurements obtained from the same patient. Differences in motion amplitude between the laterality groups (right vs. left breast) were assessed using the Mann–Whitney U test for independent, non-normally distributed samples. Correlations between motion magnitude and continuous variables (body mass index [BMI] and age) were evaluated using Spearman’s rank correlation coefficient, which does not assume linearity or normality. Multivariable linear regression was performed to identify the independent predictors of the 3D vector magnitude, with age, BMI, and laterality as covariates; the 3D magnitude was approximately normally distributed (Shapiro–Wilk, *p* = 0.077), satisfying the assumptions of the regression model. BMI was missing for 6 of the 100 patients; these patients were excluded only from the BMI-related correlation and regression analyses (available-case analysis, *n* = 94), whereas all other analyses included the full cohort (*n* = 100). To evaluate the robustness of our findings to this missingness, we performed a sensitivity analysis comparing the multivariable regression results between the available-case approach (*n* = 94) and mean imputation of BMI (*n* = 100); the direction, magnitude, and significance of all predictors were consistent between the two approaches, and the non-parametric between-group comparisons yielded conclusions concordant with their parametric counterparts. A two-tailed *p*-value < 0.05 was considered statistically significant. Statistical analyses were performed using Stata/SE version 18.5 (StataCorp LLC, College Station, TX, USA).

## 3. Results

### 3.1. Patient Characteristics

One hundred patients with BCS were included in the analysis. The patient characteristics are summarized in Table 1. The median patient age was 57 years (range, 29–81 years). Fifty-one patients (51%) had left-sided breast cancer, and 49 patients (49%) had right-sided breast cancer. The mean BMI was 23.9 ± 3.4 kg/m^2^ (available in 94 patients). Mean 3D vector motion was similar across age groups (29–44 years, 2.07 ± 0.65 mm, *n* = 14; 45–60 years, 1.96 ± 0.70 mm, *n* = 49; 61–81 years, 2.00 ± 0.66 mm, *n* = 37), with no significant difference among groups (Kruskal–Wallis test, *p* = 0.768).

### 3.2. CTV Center Displacement 

The CTV center displacement in each direction and the magnitude of the 3D vector are summarized in Table 2. The mean displacement was 0.94 ± 0.52 mm in the LR, 1.29 ± 0.59 mm in the AP, and 1.00 ± 0.51 mm in the SI directions. The mean 3D vector magnitude was 1.99 ± 0.68 mm, with a maximum of 3.51 mm (range, 0.70–3.51 mm).

**Figure 2:**
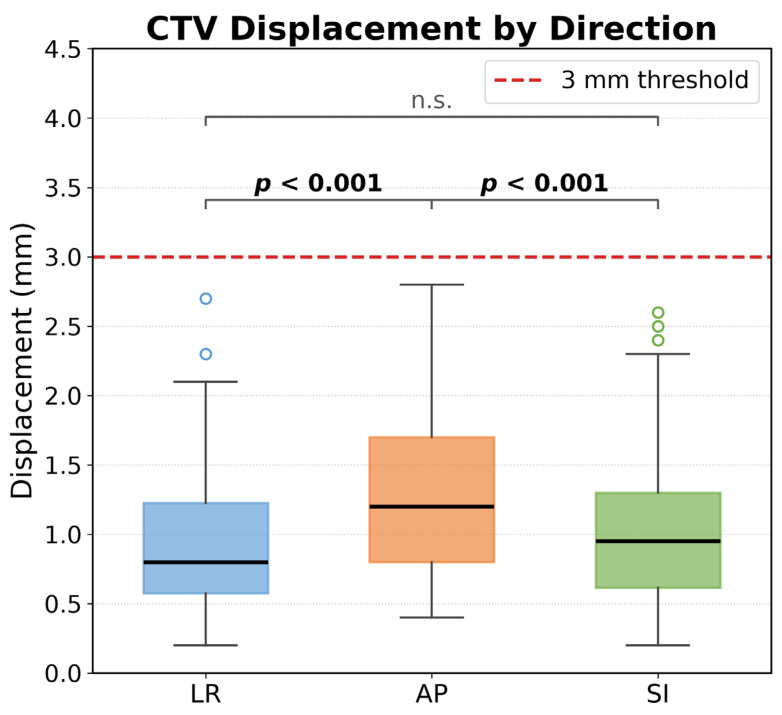
Box plot showing the distribution of CTV center displacement amplitude in the lateral (LR), anteroposterior (AP), and superoinferior (SI) directions.

Pairwise comparison revealed that the AP direction exhibited significantly greater displacement than the LR (*p* < 0.001) and SI directions (*p* < 0.001), while no significant difference was observed between the LR and SI directions (*p* = 0.274) (Figure 2).

None of the patients demonstrated displacement exceeding 3 mm in any direction. In terms of 3D vector magnitude, 95% of the patients had motions exceeding 1 mm, 48% exceeded 2 mm, and only 11% exceeded 3 mm. None of the patients exhibited a 3D vector magnitude exceeding 5 mm.

### 3.3. Association with Patient Characteristics

**Figure 3:**
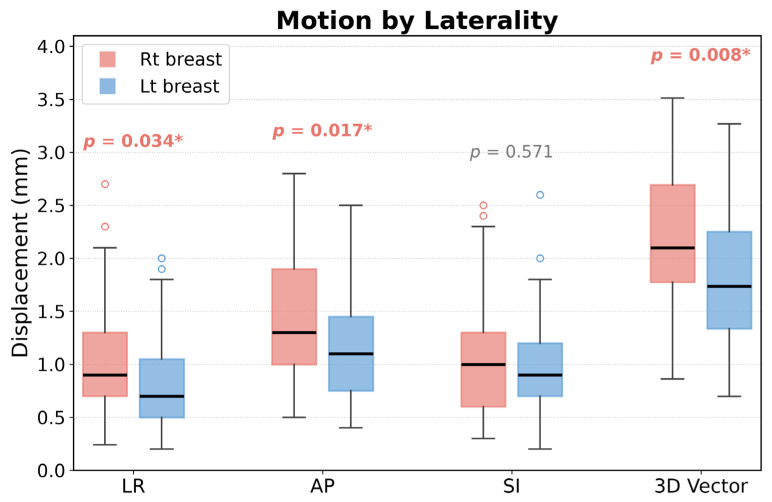
Box plots comparing CTV displacement between the right and left breast groups for each direction and the 3D vector magnitude. Asterisks (*) denote a statistically significant difference between the right- and left-breast groups (Mann–Whitney U test, *p* < 0.05).

The CTV center displacement in each direction and the 3D vector magnitude according to laterality are summarized in Table 3. Subgroup analysis by laterality revealed that patients with right-sided breast cancer exhibited significantly greater 3D vector magnitude compared to patients with left-sided disease (2.17 ± 0.66 mm vs. 1.82 ± 0.65 mm, *p* = 0.008) (Figure 3). The difference was most pronounced in the AP direction (1.42 ± 0.58 mm vs. 1.17 ± 0.58 mm, *p* = 0.017).

**Figure 4:**
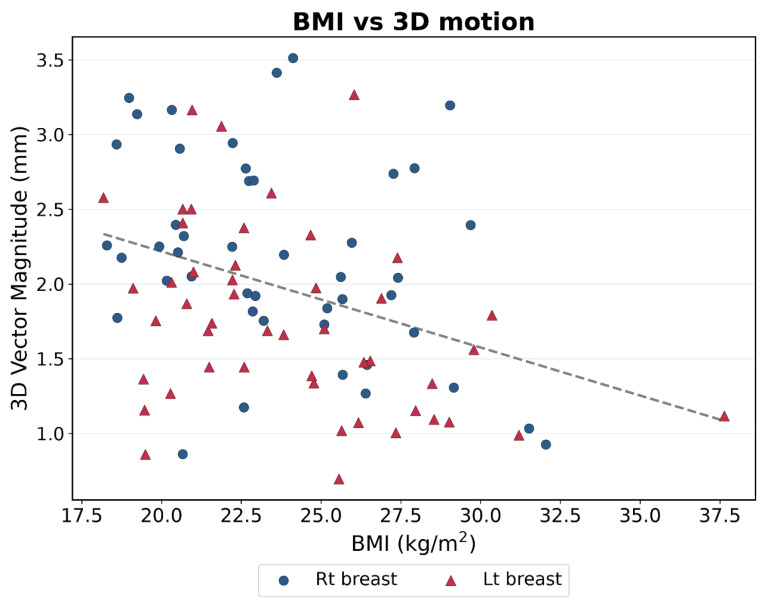
Scatterplot of BMI versus 3D vector magnitude.

Spearman correlation analysis demonstrated a significant negative correlation between BMI and the 3D vector magnitude (ρ = −0.348, *p* < 0.001) (Figure 4). This correlation was most prominent in the AP direction (ρ = −0.380, *p* < 0.001). When patients were dichotomized by BMI < 25 versus ≥ 25 kg/m^2^, the lower BMI group showed significantly greater 3D motion (2.16 ± 0.63 mm vs. 1.77 ± 0.68 mm, *p* = 0.003). Age showed no significant correlation with motion in any direction (Spearman’s ρ = 0.04, *p* = 0.68 for LR; ρ = −0.09, *p* = 0.40 for AP; ρ = 0.11, *p* = 0.30 for SI; and ρ = 0.01, *p* = 0.91 for the 3D vector magnitude), consistent with the multivariate analysis in which age was not an independent predictor (β = 0.006, *p* = 0.292; Table 4).

Multivariate linear regression analysis identified BMI (β = −0.064, *p* < 0.001) and laterality (right breast, β = 0.416, *p* = 0.001) as independent predictors of 3D vector magnitude, but age was not a significant predictor (β = 0.006, *p* = 0.292) (Table 4).

### 3.4. Internal Margin for Respiratory Motion

The internal margins calculated using the Van Herk formula are listed in Table 5. The margins were 1.02 mm in the LR direction, 1.24 mm in the AP direction, and 1.03 mm in the SI direction. The AP direction required the largest margin, which is consistent with the largest motion observed in this direction. When analyzed by laterality, patients with right-sided breast cancer required slightly larger margins (LR: 1.13, AP: 1.26, SI: 1.08 mm) than patients with left-sided breast cancer (LR, 0.88; AP, 1.19; SI, 0.99 mm).

## 4. Discussion

In this study, we quantitatively assessed respiratory-induced CTV motion during postoperative whole-breast RT in 100 patients with BCS using 4DCT. Our findings demonstrated that the CTV center displacement was consistently small in all directions, with mean amplitudes of less than 1.3 mm and maximum values not exceeding 3 mm in any individual direction. These results provide robust quantitative evidence supporting the continued use of free-breathing CT for simulations in routine whole-breast RT planning.

A notable finding of this study was that the AP direction exhibited the largest motion amplitude, significantly exceeding both the LR and SI directions. This pattern differs from that typically observed in lung cancer, where the SI direction dominates due to the direct influence of diaphragmatic excursion [6,7]. In breast cancer, the chest wall predominantly moves in the AP direction as the rib cage expands and contracts during respiration, which may explain the AP-dominant motion pattern observed in our cohort. This directional pattern is compatible with previous 4DCT and chest wall motion studies of breast RT [16,19]. Understanding this directional pattern has practical implications for treatment planning; the AP direction deserves particular attention when considering margin design, and techniques such as skin flash or virtual bolus in IMRT planning should account for residual respiratory motion in this direction [21,22].

The association between laterality and magnitude of motion is an interesting finding. The right breast showed significantly larger 3D motion than the left breast, particularly in the AP direction. Several anatomical mechanisms may contribute to this asymmetry, although they remain speculative and were not directly evaluated in the present study. One hypothesis relates to the liver and the right hemidiaphragm: the liver, a large solid organ located beneath the right hemidiaphragm, could transmit diaphragmatic excursion more effectively to the right chest wall, and the right hemidiaphragm has been reported to sit slightly higher with a somewhat greater excursion amplitude than the left. Additionally, the left lung is inherently smaller than the right owing to the cardiac notch, which may reduce the left-sided contribution to tidal volume and thereby limit left chest-wall expansion during inspiration. These proposed mechanisms should be regarded as hypothesis-generating only; the precise basis for the laterality-dependent difference cannot be established from the present data, and dedicated biomechanical and imaging studies are warranted before any causal interpretation is drawn. While the underlying mechanism remains speculative, the clinical relevance of right-sided respiratory anatomy is reflected in dosimetric studies showing that breath-hold during right-breast irradiation can meaningfully reduce liver and lung doses [23].

The negative correlation between BMI and CTV motion is a clinically relevant observation. Patients with higher BMI demonstrated smaller respiratory-induced breast motion, which likely reflects the dampening effect of subcutaneous adipose tissue on chest wall excursion. In multivariate analysis, BMI remained an independent predictor of motion magnitude, even after adjusting for age and laterality. This finding is consistent with physiological studies showing that obesity is associated with reduced chest wall compliance and tidal volume, resulting in smaller respiratory excursions [24,25]. From a clinical perspective, this suggests that leaner patients, who may paradoxically be perceived as having a less complex anatomy for treatment planning, may actually exhibit a relatively larger respiratory motion, although the absolute magnitude remains clinically small.

Although the Van Herk margin formula was originally derived in the context of patient setup errors, its underlying principle—that a target should be encompassed with adequate probability given the systematic (Σ) and random (σ) components of geometric uncertainty—is independent of the physical source of that uncertainty. We therefore applied this framework specifically to the respiratory component of CTV motion, treating the inter-patient variability in mean breathing position as the systematic component and the intrafractional respiratory excursion as the random component. A comparable application of the Van Herk formalism to derive individualized margins from breast target motion has been reported in postoperative breast cancer patients [26], supporting the validity of this approach. We emphasize that the resulting value represents an internal margin attributable to respiration alone, rather than a complete CTV-to-PTV margin; in clinical practice, setup errors and other geometric uncertainties must be combined with this respiratory component to derive the total margin. By isolating the respiratory contribution, our analysis quantifies the extent to which breathing motion accounts for the conventional margin, which was the specific aim of this study.

The calculated internal margins using the Van Herk formula were approximately 1.0–1.2 mm in all directions, with the AP direction requiring the largest margin of 1.24 mm. These values are substantially smaller than the representative clinical CTV-to-PTV margins used in breast RT, including the 5 mm margin adopted in contemporary ultrahypofractionated protocols [27], and those reported in individualized margin-assessment studies [10,26]. Although the van Herk formalism was introduced in 2000, it remains the standard population-based margin recipe in contemporary practice and continues to be applied to motion-inclusive and individualized margin estimation, including in breast RT [26]; it nonetheless assumes approximately Gaussian-distributed errors and a specific dose-coverage criterion, and a formalism dedicated to respiratory-amplitude-derived internal margins has not been extensively validated, so our estimates should be interpreted with these assumptions in mind. This indicates that respiratory motion contributes to only a limited portion of the total margin, with the remainder attributable to other sources of uncertainty, including setup error, interfractional positional variation, and delineation uncertainty. Accordingly, our data suggest that dedicated respiratory motion management techniques such as respiratory gating or breath-holding may not be routinely necessary solely for target coverage in standard whole-breast RT, although they remain valuable for reducing cardiac and lung doses, particularly in left-sided breast cancer [28,29,30].

Our findings are consistent with those of previous studies. Mankinen et al. [15] reported that a respiratory motion of less than 5 mm did not result in clinically significant changes in the planned whole-breast irradiation dose for volumetric modulated arc therapy. Choi et al. [13] demonstrated using a 3D-printed dynamic phantom that the respiratory motion during breast IMRT had a negligible dosimetric effect at amplitudes below 5 mm. Wang et al. [17] found that the CTV volume variance during respiration was very small in patients with forward-planned IMRT. The present study extends these findings by providing comprehensive motion characterization in a larger cohort of 100 patients with an analysis of associated clinical factors.

Several studies have raised concerns that respiratory motion and beam modulation during IMRT delivery could theoretically degrade dose distribution [11,12,13]. However, the motion amplitudes observed in the present study are well below the range reported to cause clinically meaningful dosimetric perturbations. Choi et al. [12] demonstrated that breast IMRT plans remained robust against respiratory motion during tidal breathing, with the main dosimetric concern being the tumor bed simultaneous integrated boost rather than the whole-breast target. Given the small motion magnitudes in our cohort, the interplay effect is unlikely to be a major clinical concern in routine whole-breast IMRT.

This study has several strengths. First, the sample size of 100 patients is one of the largest reported for this type of analysis, providing robust estimates of motion distribution and reliable subgroup analyses. Second, the use of 10 respiratory phases allowed for a comprehensive characterization of the full respiratory cycle, rather than relying on only two extreme phases. Third, the inclusion of both lateralities and the analysis of BMI as a continuous variable provide novel insights into the factors affecting breast CTV motion.

However, this study had some limitations. First, we measured only the displacement of the CTV center (center of mass) and did not assess volumetric changes in the CTV shape or the formation of an internal target volume. CTV deformation during respiration, although expected to be minimal in the breast, can provide additional information regarding motion patterns. Second, we did not perform a dosimetric analysis to directly evaluate the impact of the observed motion on the treatment plan quality. It is important to note that a small geometric motion magnitude does not necessarily equate to a negligible dosimetric effect. In particular, for highly modulated techniques such as IMRT and volumetric-modulated arc therapy, the interplay between respiratory motion and the moving multileaf collimator can produce dose deviations that are not predicted by geometric displacement alone. Therefore, although the millimetric CTV motion observed here is well within the conventional 5 mm CTV-to-PTV margin from a purely geometric standpoint, the dosimetric robustness of treatment plans under respiratory motion should be confirmed in a dedicated study incorporating dose recalculation across respiratory phases and accounting for delivery-technique-specific interplay effects; such an analysis was beyond the scope of the present study, which was designed to quantify the geometric motion of the breast CTV. Third, BMI data were unavailable for six patients, although a sensitivity analysis indicated that this did not materially affect the results. Fourth, all contours were delineated by a single observer, and intra- or interobserver reproducibility of the delineation was not formally assessed, which may have introduced a degree of measurement variability. Fifth, individual respiratory frequency and regularity were not systematically recorded for retrospective analysis; as all scans were acquired under free-breathing conditions using standard institutional protocols, patients with irregular breathing were managed according to routine clinical practice. Finally, our findings are specific to postoperative whole-breast irradiation following breast-conserving surgery and should not be extrapolated to other clinical scenarios. Settings such as regional nodal irradiation, simultaneous integrated boost, ultrahypofractionated regimens, prone-position treatment, and postmastectomy chest wall irradiation involve different target geometries, motion characteristics, or dose gradients, and would require dedicated evaluation. In addition, our measurements captured intrafractional motion at a single time point (simulation), and the reproducibility of respiratory patterns across the entire treatment course was not assessed.

## 5. Conclusions

Respiratory-induced CTV motion during postoperative whole-breast RT after BCS was small, with mean amplitudes of less than 1.3 mm and the AP direction was the dominant axis of motion. No patient exhibited displacements exceeding 3 mm in any direction. BMI and laterality were identified as independent predictors of motion magnitude, with higher BMI and left-sided tumors associated with smaller motion. The calculated internal margins for respiratory motion were approximately 1 mm, indicating that a conventional 5 mm CTV-to-PTV margin is likely sufficient to compensate for respiratory motion. These findings have direct implications for routine clinical practice: they support the continued use of free-breathing CT simulation for whole-breast RT and suggest that routine 4DCT acquisition or active respiratory motion management is unlikely to be necessary for adequate target coverage in this setting, thereby simplifying workflow and reducing simulation time and resource use. Because higher BMI and left-sided tumors were associated with smaller motion, patients with lower BMI or right-sided tumors may warrant closer attention when motion is a concern, although the absolute magnitudes remain small. These conclusions apply specifically to supine, free-breathing postoperative whole-breast RT after BCS. Future work should extend these findings through dedicated dosimetric robustness analyses incorporating dose recalculation across respiratory phases and delivery-technique-specific interplay effects. Further studies should also evaluate other clinical contexts such as regional nodal irradiation, simultaneous integrated boost, ultrahypofractionation, and prone-position treatment and prospectively assess the inter-fraction reproducibility of respiratory motion across the treatment course.

## Figures and Tables

**Table 1:** Patient Characteristics.

Variables	
Age (years), median (range)	57 (29–81)
Age group, mean 3D motion (mm)	
29–44 (*n* = 14)	2.07 ± 0.65
45–60 (*n* = 49)	1.96 ± 0.70
61–81 (*n* = 37)	2.00 ± 0.66
BMI (kg/m^2^), mean ± SD	23.9 ± 3.4
Laterality	
Right breast	49 (49%)
Left breast	51 (51%)

SD, standard deviation; BMI, body mass index; 3D, three-dimensional.

**Table 2:** CTV Center Displacement Amplitude.

Direction	Mean ± SD (mm)	Median (mm)	Range (mm)	IQR (mm)	>2 mm *n* (%)
LR (X)	0.94 ± 0.52	0.80	0.2–2.7	0.57–1.23	3 (3.0%)
AP (Y)	1.29 ± 0.59	1.20	0.4–2.8	0.80–1.70	13 (13.0%)
SI (Z)	1.00 ± 0.51	0.95	0.2–2.6	0.61–1.30	5 (5.0%)
3D vector	1.99 ± 0.68	1.96	0.7–3.5	1.46–2.40	48 (48.0%)

SD, standard deviation; IQR, interquartile range; LR, left–right; AP, anteroposterior; SI, superoinferior.

**Table 3:** CTV Displacement by Laterality.

Direction	Right (*n* = 49) Mean ± SD (mm)	Left (*n* = 51) Mean ± SD (mm)	*p*-Value
LR (X)	1.06 ± 0.57	0.82 ± 0.44	0.034
AP (Y)	1.42 ± 0.58	1.17 ± 0.58	0.017
SI (Z)	1.04 ± 0.54	0.97 ± 0.49	0.571
3D vector	2.17 ± 0.66	1.82 ± 0.65	0.008

SD, standard deviation, LR = left–right AP, anterior–posterior; SI, superoinferior.

**Table 4:** Multivariate Linear Regression Analysis for 3D Vector Magnitude.

Variable	Coefficient (β)	SE	95% CI	*p*-Value
Age (years)	0.006	0.005	−0.005, 0.016	0.292
BMI (kg/m^2^)	−0.064	0.017	−0.097, −0.030	<0.001
Laterality (Right)	0.416	0.125	0.168, 0.663	0.001

SE, standard error; CI, confidence interval; BMI, body mass index.

**Table 5:** Internal Margin for Respiratory Motion (Van Herk Formula).

Direction	Σ (mm)	σ (mm)	Margin (mm) (2.5Σ + 0.7σ)
LR (X)	0.26	0.53	1.02
AP (Y)	0.30	0.71	1.24
SI (Z)	0.26	0.57	1.03

LR, left–right; AP, anteroposterior; SI, superoinferior.

## Data Availability

The data that support the findings of this study are available upon request from the corresponding author. The data are not publicly available due to privacy or ethical restrictions.

## Abbreviations

The following abbreviations are used in this manuscript:

3D	three-dimensional
4DCT	four-dimensional computed tomography
AP	anteroposterior
BCS	breast-conserving surgery
BMI	body mass index
CRT	conformal radiotherapy
CT	computed tomography
CTV	clinical target volume
IMRT	intensity-modulated radiotherapy
IQR	interquartile range
IRB	institutional review board
LR	lateral
PTV	planning target volume
RT	radiotherapy
SI	superoinferior
